# Characterization of novel, recurrent genomic rearrangements as sensitive MRD targets in childhood B-cell precursor ALL

**DOI:** 10.1038/s41408-019-0257-x

**Published:** 2019-11-29

**Authors:** Udo zur Stadt, Malik Alawi, Manuela Adao, Daniela Indenbirken, Gabriele Escherich, Martin A. Horstmann

**Affiliations:** 1 Department of Pediatric Hematology and Oncology, University Medical Center Hamburg, 20246 Hamburg, Germany; 2 Bioinformatics Core Facility, University Medical Center Hamburg, 20246 Hamburg, Germany; 3 Heinrich Pette Institute, Leibniz-Institute for Experimental Virology, 20251 Hamburg, Germany; 4 Research Institute Children’s Cancer Center Hamburg, 20251 Hamburg, Germany

**Keywords:** Cancer genetics, Cancer, Cancer genetics, Cancer

## Abstract

B-cell precursor (BCP) ALL carry a variety of classical V(D)J rearrangements as well as genomic fusions and translocations. Here, we assessed the value of genomic capture high-throughput sequencing (gc-HTS) in BCP ALL (*n* = 183) for the identification and implementation of targets for minimal residual disease (MRD) testing. For TRδ, a total of 300 clonal rearrangements were detected in 158 of 183 samples (86%). Beside clonal Vδ2-Dδ3, Dδ2-Dδ3, and Vδ2-Jα we identified a novel group of recurrent Dδ-Jα rearrangements, comprising Dδ2 or Dδ3 segments fused predominantly to Jα29. For IGH-JH, 329 clonal rearrangements were identified in 172 of 183 samples (94%) including novel types of V(D)J joining. Oligoclonality was found in ~1/3 (*n* = 57/183) of ALL samples. Genomic breakpoints were identified in 71 BCP-ALL. A distinct MRD high-risk subgroup of IGH-V(D)J-germline ALL revealed frequent deletions of IKZF1 (*n* = 7/11) and the presence of genomic fusions (*n* = 10/11). Quantitative measurement using genomic fusion breakpoints achieved equivalent results compared to conventional V(D)J-based MRD testing and could be advantageous upon persistence of a leukemic clone. Taken together, selective gc-HTS expands the spectrum of suitable MRD targets and allows for the identification of genomic fusions relevant to risk and treatment stratification in childhood ALL.

## Introduction

Precise monitoring of minimal residual disease (MRD) during the first weeks of treatment supports decisions on escalation or de-escalation of therapy in patients with acute lymphoblastic leukemia (ALL)^1,2^. The treatment response measured by sensitive MRD techniques at the end of induction therapy (EOI) has been shown to be one of the strongest parameters for risk stratification. Initially, Southern blot and semiquantitative PCR-based methodologies were applied which demonstrated frequent clonal rearrangements of *IGH* and *TR-δ* segments in BCP-ALL^3^. Current technological approaches to the measurement of MRD comprise quantitative real-time PCR or multicolor flow cytometry allowing for reproducible quantification of residual leukemic cells with a sensitivity of one leukemic cell among 10,000 normal cells (1 × 10^−4^)^4,5^. In order to trace leukemic cells in a clinical setting, the rapid identification of suitable markers is essential for their subsequent quantification. A PCR-based screening method is currently implemented as the standard procedure for the detection of patient-specific V(D)J rearrangements^6^. With the establishment of recent, PCR-based high-throughput sequencing techniques (HTS) the analytical process of identification and characterization of patient-specific V(D)J rearrangements largely relies on bioinformatics^7–12^. As an alternative to PCR-based amplification of target sequences, HTS of directly captured genomic fragments has recently been employed for the identification of rearrangements^13,14^. The advantage of the latter approach is the potential identification of unknown fusion sequences, i.e., rare or unusual V(D)J rearrangements or chromosomal translocations not confined to IG or TR genes which could be used for MRD diagnostics and/or targeted therapy of actionable fusion proteins, such as *ABL* or *JAK2* class fusions^15,16^.

Here we show that genomic capture high-throughput sequencing (gc-HTS) identified a novel type of recurrent clonal V(D)J TRδ Dδ-Jα rearrangements as well as diverse genomic fusion breakpoints which can be used as sensitive and specific markers in clinical diagnostics of MRD for risk-adapted treatment stratification and targeted intervention. ALL samples devoid of detectable IGH-V(D)J rearrangements were associated with high levels of MRD at EOI and often featured genomic fusion events, such as *DUX4-IGH and EPOR-IGH*.

## Patients, material, and methods

Patients involved in this study were enrolled in COALL03-07 and COALL08-09 multicenter trials (www.clinicaltrials.gov: GPOH-COALL08-09 EU-21076/NCT01228331) except for *BCR-ABL1* positive ALL patients who were treated according to the international EsPhALL trial (EudraCT 2004-001647-30 and *clinicaltrials.gov Identifier: 00287105*)^17^. Patient samples were obtained after written informed consent of the patients’ parents or legal guardians and with approval by institutional ethics boards (PVN3409; EudraCT-Nr: 2009-012758-18).

**Fig. 1:**
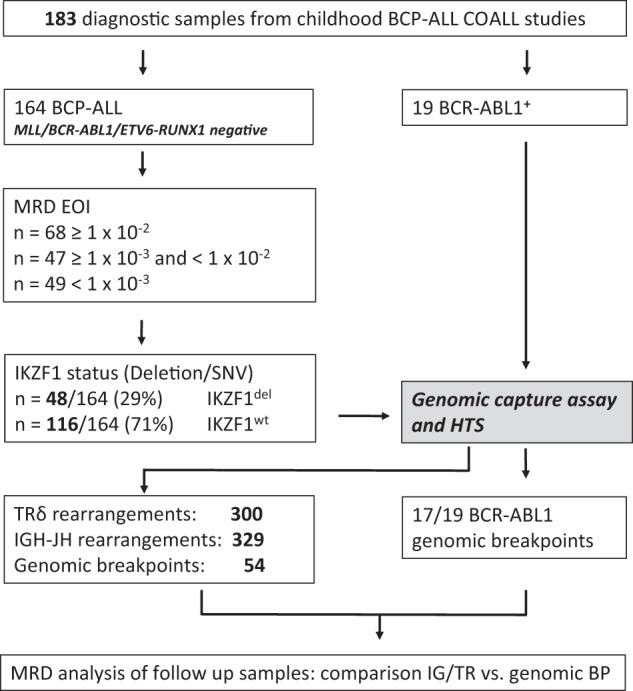
Experimental flow diagram. A total number of 183 BCP-ALL cases were analyzed with the gc-HTS approach. Nineteen of them had a known *BCR-ABL1* fusion identified by either FISH or molecular genetics. The 164 remaining cases were further divided in three subgroups according to the MRD status at EOI therapy. *IKZF1* deletions/mutations were detected by MLPA or Sanger sequencing. Genomic fusion breakpoints were used for quantification of follow-up samples by RQ-PCR.

All gc-HTS analysed cases (*n* = 183) were diagnosed as B-precursor ALL devoid of *KMT2A* and *ETV6-RUNX1* rearrangements including a subgroup of *n* = 19 BCP-ALL carrying a known *BCR-ABL* rearrangement as a positive control for precise breakpoint characterization. For identification of *IKZF1* and *P2RY8-CRLF2* (PAR1) deletions multiplex ligation-dependent probe amplification (Salsa MLPA kit P335; (MRC Holland)) was used. MRD-negative or weakly positive ALL (minimum quantitative threshold < 1 × 10^−4^) exhibiting genetic low-risk features were excluded from this study except *n* = 7 subsequently relapsing ALL (*n* = 3 MRD-negative and *n* = 4 weakly positive cases). Selected ALL samples were grouped into three different quantitative categories based on their EOI MRD levels (≥1 × 10^−2^ [MRD^very_high^], ≥1 × 10^−3^ [MRD^high^], and <1 × 10^−3^ [MRD^moderate^]) as indicated in Fig. 1.

Diagnostic samples were analyzed by gc-HTS to detect clonal *TRδ* or *IGH*-JH rearrangements at diagnosis of ALL in comparison to a standard PCR-based approach. In parallel, we analyzed gene-fusion events targeting cytokine receptors or kinases as observed in Ph-like ALL^15,16^. Methodological details and the open source software Segemehl for detection of gene fusions have been previously described^13,18^. Supplemental Table 1 defines the exact chromosomal coordinates of all captured genomic regions. Novel gene fusions detected by gc-HTS were confirmed by PCR and Sanger sequencing. Quantification of classical V(D)J rearrangements as well as for non V(D)J rearrangements/genomic fusion breakpoints (that include large intra-chromosomal deletions like EBF1-PDGFRß or chromosomal translocation breakpoints) followed EUROMRD guidelines^4^. Sequence data have been submitted to the European Nucleotide Archive (ENA) and they are publicly available at: http:/www.ebi.ac.uk/ena/data/view/PRJEB35051. Sample identifier are available upon request.

## Results

In this study we sought to explore the potential of gc-HTS to concurrently identify clonal rearrangements and genomic breakpoints for the sensitive and specific detection of minimal residual disease in B-cell precursor ALL (Fig. 1).

### TRδ clonality

**Fig. 2:**
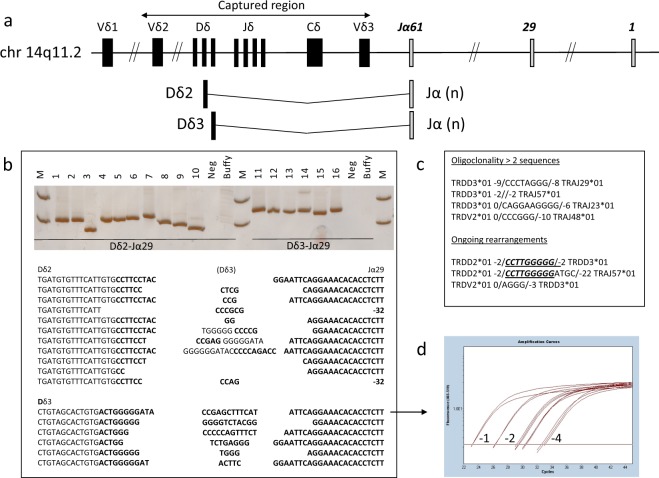
Identification of TRδ clonality. **a** Schematic view of the captured TRδ region. Novel incomplete TRδ rearrangements were detectable fused to one of the 61 Jα segments. **b** Validation of either Dδ2 or Dδ3–Jα29 rearrangement in selected cases by PCR and subsequent Sanger sequencing. **c** Several cases showed either oligoclonality with >2 sequences or ongoing rearrangements within the TRδ locus. A number before/after brackets describe the number of nucleotides deleted within the corresponding germline segment. **d** Real-time quantitative PCR was performed in a selected ALL sample with Dδ3-Jα29 rearrangement; quantitative range (QR) 1 × 10^−4^.

First, we examined the *TRδ* gene locus on chromosome 14p12 using 196 capture probes that cover a 42 kb region between Vδ2 and Vδ3 (Fig. 2a and Supplemental Fig. 1). This region is commonly involved in clonal rearrangements in ALL^19,20^. We identified clonal *TRδ* rearrangements in 158 out of 183 BCP-ALL samples (86%), the majority of which involved Vδ2 joined to Dδ3 or to Jα as shown in Table 1. The conception of the applied gc-HTS approach and bioinformatics methodologies allowed for the identification of previously unrecognized Jα-rearrangements fused either to Dδ2- or to Dδ3-segments. Beside well-characterized clonal Vδ2-Jα joinings, we identified 63 rearrangements of Jα segments to Dδ2 (*n* = 39) or Dδ3 (*n* = 24) in 53 of 183 samples (29%), with Jα29 being the most common single *TRJα* fragment (*n* = 25). The frequency of these rearrangements substantially exceeded the previously reported prevalence in gene-specific PCR-based analyses^20,21^. To revalidate NGS-derived *TRδ* rearrangements by Sanger sequencing, amplicons were generated by a PCR strategy positioning forward primers in intronic Dδ2 and Dδ3 regions combined with a Jα29 specific reverse primer (Fig. 2b). Since the predictive value of MRD testing is dependent on the (sub)clonality of targeted rearranged sequences, we determined the number of *TRδ* rearrangements per sample. *TRδ* oligoclonality defined as greater than two sequences per ALL sample was observed in 34 of 158 positive specimens, the frequency of which is in accordance with previous reports based on Southern Blot or PCR-based methods^19^. Several ALL cases demonstrated clonally related *TRδ*-rearrangements with regard to their N-region insertions, probably due to ongoing recombination from *Dδ2*- *Dδ3* to *Dδ2-Jα* segments (Fig. 2c)^22^. The knowledge of these additional clones is of importance for subsequent MRD testing as oligoclonal TRδ targets generally have a lower stability compared to clonal rearrangements.

**Table 1:** Frequency of clonal IGH/TRδ rearrangements.

Type of rearrangement	number of positive rearrangements	number of positive samples (Σ 183)
*TRδ*	300	158 (86%)
Vδ2-Dδ3	118	88 (48%)
Dδ2-Dδ3	34	29 (16%)
Vδ2-Jα*	85	71 (39%)
Dδ - Jα*	63	53 (29%)
• Dδ2- Jα*	39	
• Dδ3- Jα*	24	
Vδ-Jδ	2	2 (1%)
TRδ oligoclonality (>2)	111	34 (19%)
*IGH*	329	172 (94%)
V(D)J	254	157 (86%)
DJ	75	56 (31%)
IGH oligoclonality (>2)	66	19 (10%)

Jα*—one of the Jα01 to Jα61 segments, with the majority of cases positive for Jα29

### IGH clonality

**Fig. 3:**
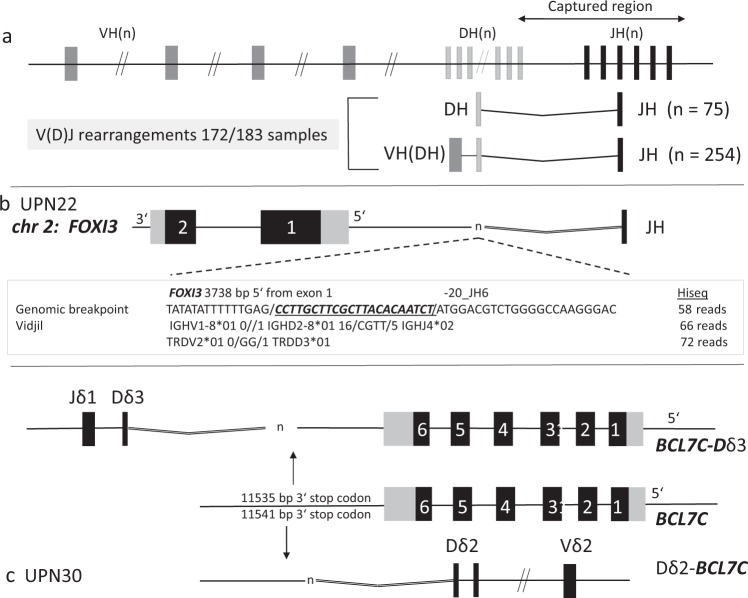
Schematic view of the captured *IGH-JH* area on chromosome 14q. **a** At least one clonal *DH-JH* or *VH(DH)JH* rearrangement was identified in 172/183 cases indicative of an IGH germline status in the remaining 11 cases. **b** A *FOXI3-JH6* genomic fusion breakpoint was identified in case ALL UPN22. Fifty eight breakpoint spanning reads covered the breakpoint and for the same sample VIDJIL identified two different clonal rearrangements with 66 and 72 reads, respectively. **c** For UPN30 two genomic fusion breakpoints (*BCL7C-Dδ3* and *Dδ2-BCL7C*) were sequenced. The different breakpoints were located 11.5 Kb downstream of the *BCL7C* stop codon.

In BCP-ALL, clonal *IGH* rearrangements are very common and the most suitable and sensitive marker for MRD detection^3,6^. To examine the *IGH-JH* locus on chromosome 14q32 by gc-HTS we selected 184 capture probes comprising 22 kb of genomic sequence (Fig. 3a). *IGH*-V(D)J-joining was identified in 172 of the 183 samples (94%) with 329 clonal sequences including complete V(D)J-rearrangements as well as incomplete DJ-joining. Hence, gc-HTS identified *n* = 11 ALL samples truly devoid of *IGH*-V(D)J rearrangements (Table 2 and Table 3). Oligoclonal *IGH*-V(D)J rearrangements were identified in *n* = 19 cases (10%).

**Table 2:** Frequency of genomic rearrangements within the three different MRD groups.

MRD value EOI/Genomic rearrangement	≥1 × 10^−2^	<1 × 10^−2^	<1 × 10^−3^
	(*n* = 68)	(*n* = 47)	(*n* = 49)^a^
No rearrangement	37	39	36
DUX4-IGH	6	3	1
CRLF2-IGH	6	–	–
EBF1-PDGFR-ß	5	–	–
EPOR-IGH	5	–	1^b^
PAR1-Del	4	3	3 (2^c^)
other	6	2	8
	31 (46%)	8 (17%)	13 (26%)

*EOI* end of induction treatment

^a^This group included seven selected relapse cases with weakly positive or negative MRD

^b^Patient had a relapse and was initially grouped as MRD-EOI “positive, not quantifiable”, but new MRD marker would re-assign this patient to the second group

^c^Two out of the three cases were included because of a relapse and “positive, not quantifiable” MRD

**Table 3:** Basic clinical data of samples without any IGH_V(D)J rearrangement.

Age at Dx (y)	MRD_EOI	IGH_V(D)J	IKZF1	UPN	Fusion	Response EOI	Therapy
17	9.00E-03	–	del 1–8	37	BCR-ABL1	yes	Tx
17.5	9.00E-02	–	del 4–7	21	CRLF2-IGH	LateResp	Tx
11	9.00E-02	–	del 2–8	1	DUX4-IGH	LateResp	Tx
16	2.00E-02	–	wt	10	DUX4-IGH	pesistent MRD	Tx
5	2.00E-03	–	del 4–7	8	DUX4-IGH	Yes	Remission
10	7.00E-02	–	wt	61	EBF-PDGFRB	LateResp	/COALL^a^
7.5	4.00E-01	–	del 2–7	12	EPOR-IGH	LateResp	TRM
13	2.00E-01	–	del 4–8	16	EPOR-IGH	LateResp	Tx
17.5	9.00E-02	–	del 2–3	13	EPOR-IGH	LateResp	Tx
16	5.00E-02	–	del 2–8	69	FOXP1-ABL1	LateResp	Tx
12	2.00E-02	–	wt	72	n.d.	LateResp	Rel/Tx 2.rem

*n.d.* not detectable, *Rel* relapse, *Rem* remission, *Tx* Hematopoietic Stem Cell Transplantation

*EOI* end of induction therapy, *TRM* treatment related mortality

^a^off protocol treatment intensification

### Gene fusions and identification of genomic breakpoints

To advance the clinical assessment of MRD further beyond *TR*/*IG* V(D)J rearrangements we explored the potential of gc-HTS to identify the genomic breakpoints of leukemia-specific gene fusions as diagnostic targets. As a positive control, we first examined breakpoint-flanking sequences in a subgroup of *BCR-ABL1* positive ALL (*n* = 19) by gc-HTS. *ABL1*-directed capturing and subsequent paired-end sequencing identified the genomic breakpoint in 17 of 19 samples at bp resolution indicating a high sensitivity and specificity of our analytical approach. As a next step *TRδ*, *IGH*, kinase and cytokine receptor genes as outlined in Supplemental Table 1 were analyzed with regard to genomic fusion breakpoints. Overall, among *n* = 183 BCP-ALL we identified *n* = 71 non-V(D)J gene-fusion events and deciphered their genomic fusion breakpoints. These mutations recurrently involved *ABL1* (*n* = 17 *BCR-ABL1*; *n* = 1 *FOXP1-ABL1*; *n* = 1 *RCSD1-ABL1*), *CRLF2* (*n* = 11 *PAR1* deletions; *n* = 5 *CRLF2-IGH*), *DUX4* (*n* = 10 *DUX4-IGH*), *EPOR* (*n* = 6 *EPOR-IGH*), *PDGFRB* (*n* = 5 *EBF1-PDGFRB*), *JAK2* (*n* = 1 *ETV6-JAK2*; *n* = 1 *BCR-JAK2*; *n* = 1 *PAX5-JAK2*) and less frequently mostly transcription-factor associated genes (Table 4 and Table 5). Among the latter group, *FOXI3* was identified as a novel, previously unrecognized fusion partner of *IGH*. The *FOXI3* gene on chromosome 2 belongs to the large family of forkhead box transcription-factor genes consisting of two exons, which encode for a 422-aa protein with activities in embryogenesis, bone modeling and potentially in carcinogenesis^23^. Recently, functional mapping of FOXI3 has revealed a nuclear localization sequence (NLS) and a C-terminal transactivation domain (TAD)^24^. We mapped the genomic breakpoint to a region 3859 bp upstream of the ATG start codon, which was fused to *IGH-JH6* suggesting an aberrant expression driven by the *IGH*-enhancer (Fig. 3b). In accordance, FOXI3 transcripts were found to be abundantly expressed in the *FOXI3-JH6* rearranged ALL in contrast to a barely discernible or absent expression in 25 randomly selected primary BCP-ALL samples (Supplemental Fig. 2).

**Table 4:**
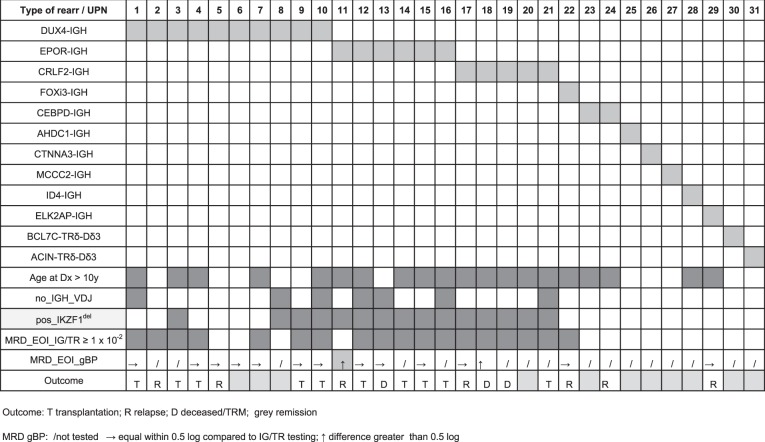
Basic clinical and molecular data of IGH and TRδ associated gene fusions.

**Table 5:**
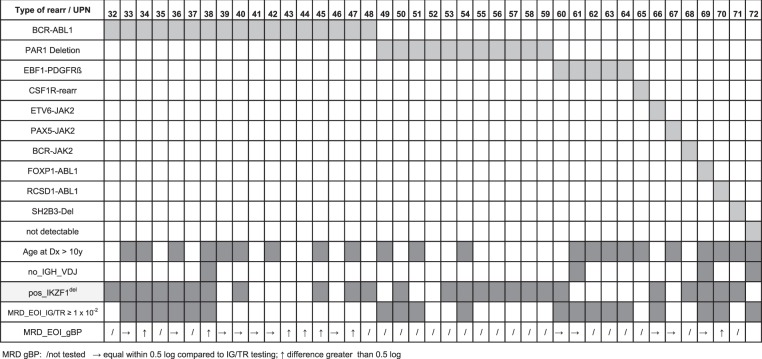
Basic clinical and molecular data of non-IGH/TRδ associated gene fusions.

Additional chromosomal rearrangements into *IGH*-JH involved *CEBPD*, *CTNNA3*, *AHDC1, MCCC2*, and *ELK2AP* (Table 4 and Table 6). We identified *CEBPD-IGH* fusions (*n* = 3) in two ALL samples from patients with Down syndrome (DS)^25,26^. Breakpoints were located in different introns of the *SPIDR* gene displaying 53 kb and 7.8 kb distance to the 3’-end of *CEBPD* at primary diagnosis (UPN23 and UPN24). The latter patient developed relapsing disease 10 years after the initial diagnosis, albeit displaying a different breakpoint 257 kb downstream of the 3’-end of *CEBPD* indicative of a different sub-clone or potentially an independent secondary leukemia. Furthermore, in UPN27 a genomic fusion of *IGH*-JH4 into the *MCCC2* gene comprising 17 coding exons on chromosome 5q13 was found with the breakpoint located in intron 16. As a consequence, *MCCC2* exon 17 and a part of intron 16 were fused to the *IGH*-JH4 region suggesting that the translocation event is non-functional or that an alternative gene further downstream is involved. Finally, in UPN29 we identified a genomic breakpoint 8994 bp 5’ of the first exon of *ELK2AP*, a processed pseudogene on chromosome 14q32^27,28^. The functional consequences of the *ELK2AP-JH1* fusion that was likely caused by an inversion affecting the IGH locus are unknown^29^. The exact genome coordinates of all novel genomic breakpoints identified here were confirmed by PCR and Sanger sequencing (Table 6).

**Table 6:** Genomic coordiates of novel genomic breakpoints.

UPN	Fusion	Gene_A	hg38	Gene_B	hg38	FSR	PCR/Seq	Comment
22	FOXI3_IGH	FOXI3_5’	chr2: 88456394	IGH_JH6	chr14:105863240	58 (H)	Yes	Dx
23	CEBPD_IGH	CEBPD_3’ (53Kb)	chr8:47684174	IGH_JH4	chr14:105864260	13 (M)	Yes	Dx
24	CEBPD_IGH	CEBPD_3’ (7Kb)	chr8:47729469	IGH_JH5-JH6	chr14:105863653	6(M)	Yes	Dx
24	CEBPD_IGH	CEBPD_3’ (257Kb)	chr8:47479748	IGH_JH5	chr14:105863864	4(M)	Yes	Relapse
25	AHDC1_IGH	AHDC1_3'	chr1:27547201	IGH_JH4	chr14:105864248	15 (H)	Yes	Dx
26	CTNNA3_IGH	CTNNA3 intron 6	chr10:67402606	IGH_JH4	chr14:105864251	22(M)	Yes	Dx
27	MCCC2_IGH	MCCC2_intron 16	chr5:71655478	IGH_JH4	chr14:105864257	73(M)	Yes	Dx
29	ELK2AP-IGH	ELK2AP_5'	chr14_105681797	IGH_JH1	chr14:105865454	168(H)	Yes	Dx (possible inversion)
29	ELK2AP-IGH	id	id	id	id	n.t.	Yes	Relapse
30	BCL7C-TRδ	BCL7C_3'	chr16:30876320	TRδ_Dδ2	chr14:22439011	99 (H)	Yes	Dx (rev)
30	BCL7C-TRδ	BCL7C_3'	chr16:30876329	TRδ_Dδ3	chr14:22449125	4(H)	Yes	Dx (forw)
31	ACIN-TRδ	ACIN1_5'	chr14:23095837	TRδ_Dδ2	chr14_22439016	44(H)	Yes	Dx
65	CSF1R-?	CSF1R_intron 11	chr5:150067115	?	chr5:92365792	36(M)	Yes	Dx (forw)
65	CSF1R-?	CSF1R_intron 11	chr5:150067680	CCNJL_intron 4	chr5:160261418	10(M)	Yes	Dx (rev)

All novel genomic breakpoints detected by gc-HTS were confirmed by PCR and Sanger Sequencing. Comment: Fusion detected at diagnosis (dx) and/or relapse

*FSR* fusion spanning reads; *(H)* HiSeq, *(M)* Miseq, *forw* forward orientation, *rev* reverse orientation

Taken together, *IGH*-associated gene fusions were detected in 31 samples, with *DUX4* rearrangements as the most frequent partner gene identified in 10 ALL cases. Genomic breakpoints were distributed either within a region 5’ upstream of *DUX4* or within the 3’-coding region in exon 1 fused to *IGH*-JH or *IGH*-DH as previously reported (Supplemental Fig. 3)^30–32^.

With regard to TRδ, we identified two previously unrecognized rearrangements into the *BCL7C* and *ACIN1* gene loci, respectively. This type of fusion, which likely places the target gene under the control of the *TRδ* enhancer/promoter, has been previously described in T-ALL^33–35^. The *BCL7C* gene on chromosome 12q24 was identified as a novel fusion partner of *TRδ* (Fig. 3c). Split-read and subsequent PCR analyses revealed that the *TRδ*-Dδ2 segment was fused to the untranslated 3’-region of *BCL7C*, whereas *TRδ*-Dδ3 is linked to a region 3’ of *BCL7C* exon 6 comprising the complete coding region of this gene (UPN30; Fig. 3c). Both breakpoints showed variable insertions of random nucleotides similar to a previously described inversion that joins *TRδ*-Dδ3 and *BCL11B*^34^. *BCL7C* belongs to the small group of *BCL7* genes, among which *BCL7A* has previously been detected in high-grade B-cell lymphoma as a partner gene in a complex chromosomal translocation t(8;14;12) that involves *c-MYC* as well as *IGH*-VH3^36^. The second rearrangement involved the *TRδ*-Dδ2 segment fused to a region immediately 5’ of the start codon of *ACIN1* located on chromosome 14q11 indicative of a non-functional break on the reverse strand (Supplemental Fig. 4; UPN 31). Of note, *ACIN1* has recently been described as a fusion partner gene of *NUTM1* in ALL^37^. The TRδ-Dδ3 related breakpoint site, which normally activates the corresponding target gene through strong TRδ enhancer elements, could not be identified. The most adjacent gene on the reverse strand belongs to the CEBP family (CEBPE), which is associated with translocations affecting the *IGH* locus on chromosome 14q32^25^.

With regard to kinase coding genes we detected single genomic breakpoints of previously described gene fusions that involve *RCSD1-ABL1*, *FOXP1-ABL1*, *BCR-JAK2*, *PAX5-JAK2*, and *ETV6-JAK2*^15,16,38^. We identified a novel breakpoint in *CSF1R* intron 11 fused to a yet undefined intergenic region on chromosome 5. This genomic fusion contains a random seven-nucleotide insertion that was not discernible in previously analyzed ALL cases indicating a specific breakpoint with yet unknown functional relevance. The reverse breakpoint was located in intron 10 fused to intron 4 of the *CCNJL* gene (Table 6; UPN65)

### Very high MRD is associated with non-V(D)J genomic rearrangements

A high burden of MRD after induction therapy (EOI) is generally associated with a poor prognosis^1,2,39^. To investigate type and prevalence of genomic rearrangements in MRD-defined very high-risk ALL we categorized *n* = 164 ALL samples into three subgroups according to their quantitative MRD EOI levels as outlined in Materials and Methods. In the MRD^very_high^ subgroup, 31 of 68 ALL samples (46%) carried genomic rearrangements compared to 17 and 26% fusion events in ALL exhibiting a moderate or high MRD burden, respectively (Table 2). All *EBF1*-*PDGFR-ß* and *CRLF2*-*IGH* rearrangement positive cases as well as five of six ALL harboring *EPOR*-*IGH* fusions were found in the MRD very high-risk group. Strikingly, six out of 10 *DUX4*-rearranged cases exhibited very high MRD levels at EOI (≥1 × 10^−2^) that led to hematopoietic stem cell transplantation in first remission. Another *DUX4*^*rearr*^ ALL (UPN5) with an MRD burden of 9 × 10^−3^ at EOI developed two relapses, finally exhibiting a phenotypical switch to the myeloid lineage but stable clonal V(D)J-rearrangements used as molecular MRD markers^40^. ALL harboring *DUX4*-rearrangements have previously been described as a prognostically favorable ALL-subgroup often containing concomitant intragenic *ERG*^*del*^ or a deregulation of *ERG* by expression of an alternatively spliced, dominant-negative ERG isoform (ERG^alt^)^31^. In our DUX4^rearr^ ALL cohort, ERG^del^ were detected in 2/10 cases by MLPA analysis, whereas ERG^alt^ was identified in 4 of 5 available *DUX4*^*rearr*^ ALL samples by isoform-specific RT-PCR in accordance with previously reported data^31^.

Among the small group of primary ALL (*n* = 7) relapsing later on with weakly positive or negative MRD at EOI analyzed in this study, a single case harbored an *EPOR-IGH* rearrangement (UPN11). Intriguingly, re-analysis of MRD in this case utilizing the *EPOR-IGH* breakpoint and a clonal Vδ2-Jα22 rearrangement as additional markers identified by gc-HTS would have resulted in an assignment to a higher risk group and more intensified treatment (Table 4).

A germline V(D)J constitution at the *IGH* locus as observed in 11 out of 183 analyzed ALL (6%) indicates a leukemic transformation at a very immature, early stage of B-cell development^3,9^. Germline *IGH*-V(D)J ALL were associated with poor treatment response, frequent *IKZF1* deletions and genomic rearrangements such as *DUX4-IGH*, *EPOR-IGH*, *BCR-ABL1*, *FOXP1-ABL1*, *EBF1-PDGFR-ß*, or a *CRLF2-IGH* (Table 3).

### Novel V(D)J and non-V(D)J genomic rearrangements are useful markers in MRD diagnostics

As outlined above we identified several novel TRδ-Jα clonal rearrangements which we sought to evaluate as optional markers for MRD diagnostics. Moreover, we established individual, patient-specific RQ-PCR assays based on genomic breakpoint sequences of non-V(D)J rearrangements. In regard to novel TRδ Dδ2- and Dδ3-specific rearrangements, we designed two RQ-PCR assays based on their germline sequences to allow for a Jα-independent quantification (Fig. 2d). In comparison, conventional and novel MRD markers showed similar sensitivities in the detection of EOI MRD presenting values deviating less than 0.5 log from each other as evaluated according to the EURO-MRD guidelines for quantification. We identified novel TRδ/α markers in almost a quarter of ALL, which could improve clinical diagnostics of MRD because of their specific amplification with a superior signal to noise ratio and a quantitative range of 1 × 10^−4^. These features render TRδ markers more suitable than other targets such as TRγ due to a low or absent background amplification.

In line with this notion, gc-HTS identified three additional MRD markers in a relapsed ALL (UPN29; Table 2 and Supplemental Table 2), which revealed slightly higher MRD values targeting the new rearrangements (Dδ2-Jα09; 5’-VH4-JH6 and *ELK2AP-JH1*) compared to routinely assessed TRß and a concomitant loss of two TRγ markers. Targeting the *IGH* locus in this sample, gc-HTS identified two unusual *IGH*-JH associated fusions not detectable by a PCR-based approach, both of which were discernible at relapse. Firstly, an IGH-JH6 segment was rearranged into an intronic region between two IGH-VH segments (7365 bp 5’ of VH7*34). Secondly, a somatic fusion event of the ETS-family pseudogene *ELK2AP* with an IGH-JH1 segment was deciphered at primary diagnosis and at relapse.

**Fig. 4:**
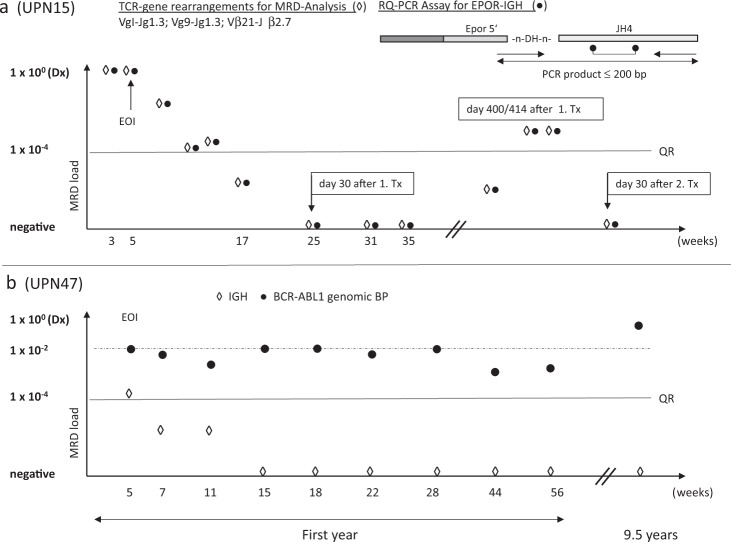
RQ-PCR assays using the genomic fusion breakpoint. **a** In sample UPN 15 conventional PCR screening identified three common TRγ/ß clonal marker used for MRD quantification; gc-HTS identified an additional *EPOR-JH4* breakpoint with a RQ-PCR QR of 1 × 10^−4^. Because of a poor response towards frontline treatment as reflected by substantial molecular residual disease the patient was transplanted in first remission, but developed a relapse one year later. Both TR- and genomic breakpoint directed analytical approaches confirmed long term molecular remission after second transplantation (>3.5 years). **b** MRD follow-up for a *BCR-ABL1*-positive patient (UPN47). The patient relapsed 10 years after first diagnosis. In contrast to IG/TR targeted MRD testing showing a rapid early response, *BCR-ABL1* genomic fusion breakpoint based RQ-PCR revealed persistently high MRD levels during the first year of treatment. At relapse, only the genomic fusion breakpoint-specific PCR showed a specific signal with loss of all previously identified IG/TR related markers.

Beside novel TR and IGH rearrangements, a total of *n* = 25 non-V(D)J rearrangements including their respective breakpoints were evaluated as diagnostic targets for MRD analysis. In contrast to the BCR-ABL1-positive ALL-subgroup which clearly exhibited breakpoint-specific signals upon remission likely originating from a rearrangement persisting in the hematopoietic stem cell population, non-V(D)J rearrangements in general revealed MRD kinetics that were very similar to conventional IG/TR-V(D)J rearrangements (Fig. 4 and Table 3)^41^.

Overall, gc-HTS alleviates the identification and implementation of genomic breakpoints and fusions as targets for MRD analysis, which might improve the monitoring of patients with a slow molecular response to chemotherapy and/or immunotherapy requiring sensitive surveillance of subclonal disease.

## Discussion

The analysis of MRD by real-time quantitative genomic PCR or multicolor flow cytometry has been firmly established as an integral part of clinical diagnostics of acute lymphoblastic leukemia^4,5^. Both methodological approaches complement each other and allow for treatment stratification in the vast majority of patients. Nevertheless, a small but significant number of patients (~5–10%) lack leukemia-specific conventional genomic V(D)J rearrangements at TR/IG loci or unambiguous cell surface epitopes preventing a reliable and sensitive measurement of MRD^42^. In addition, RQ-PCR and flow cytometry are limited in the sensitive detection of subclonal disease, which is often driven by non-V(D)J rearrangements or gene fusions not detected by conventional PCR-based MRD analyses. In line with this notion, it is generally appealing to implement predisposing or causative genetic lesions as diagnostic targets for the measurement of MRD as established for various *MLL*-gene fusions in infant ALL or, as recently published, for *ETV6-RUNX1* breakpoints^43–45^. High-throughput genome, exome and transcriptome sequencing has recently unfolded a variety of previously unrecognized genetic subtypes of B-cell precursor acute lymphoblastic leukemia^46^. Here, we present gc-HTS as an analytical approach to the combined identification of clonality and individual genomic breakpoints in B-cell precursor ALL. In accordance with PCR-based analyses, we identified classical V(D)J rearrangements at *IGH* and *TRδ* gene loci in 95% and 84% of ALL samples, respectively^6^. As an advancement, our PCR-independent approach allows for the detection of V(D)J recombinations and non-V(D)J rearrangements not discernible by PCR using pre-selected primer sets. Hence, gc-HTS substantially expands the spectrum of individual markers per ALL sample including distinct genomic aberrations, the latter of which are less prone to clonal evolution and early loss of specificity. In contrast, secondary mutational events or targets with an oligoclonal appearance such as *PAR1*- or *ERG*- deletions are less suitable for a reliable and robust MRD analysis.

In comparison, PCR-based HTS has a greater capability to detect minor V(D)J clones at diagnosis^7,47^. In a recently published study, paired analysis of diagnostic/relapse ALL samples by PCR-based HTS revealed that initial (“major”) clones with a frequency >5% or an absolute number of reads (ARC) >10,000 showed the highest stability at relapse^48^. gc-HTS does not achieve a read-depth comparable with PCR-based HTS but our preliminary data indicate a detection limit of genomic capturing for rearrangements in heterogeneous cell populations with a blast fraction of ~20%, which is sufficient for most diagnostic settings. To date, clinically relevant RQ-PCR-based MRD testing is confined to initially selected major clones. Currently, most NGS-based approaches use a nested PCR workflow for clonality detection, a strategy that requires rigorous precautions under strict GLP conditions^7,10–12,47^. By contrast, gc-HTS affixes sample-specific barcodes to the genomic fragments during the initial steps of library preparation, minimizing the risk of false-positive target assignment and contamination. In effect, the lower read-depth of gc-HTS might be compensated by the direct sequencing of enriched genomic fragments that enables us to detect rearrangements not detectable by PCR-based methods.

Beyond conventional rearrangements, previously unrecognized IG and recurrent TRδ rearrangements were deciphered, which increase the spectrum of suitable targets for clinical diagnostics of MRD. By identification of the Dδ-Jα subgroup additional targets are available for MRD testing. Nevertheless, based on these results TRδ-related oligoclonality or ongoing rearrangement patterns should be handled with caution as they can potentially result in divergent MRD values in follow-up samples. Importantly, novel previously unrecognized fusion partner genes of *IGH* were detected the function of which has yet to be defined. In line with earlier observations, we identified a subgroup of children with ALL displaying a germline *IGH* that was associated with a poor response to induction treatment and accordingly high levels of MRD^3,9^. Strikingly, this *IGH* germline ALL group exhibited a high frequency of *IKZF1* deletions and non-V(D)J rearrangements including *CRLF2-IGH*, *EPOR-IGH*, and *EBF1-PDGFRß* that can be exploited as leukemia-specific targets in clinical diagnostics of MRD. Early identification of this high-risk subgroup is a prerequisite for treatment stratification or timely intervention. Unexpectedly, the *IGH* germline ALL-subgroup also included *DUX4*-rearranged ALL lacking concurrent ERG-deletions, but some expressed the dominant-negative ERG^alt^ isoform^49^. In contrast to the previously reported favorable outcome of DUX4^rearr^-ALL we observed a remarkably poor treatment response of *DUX4*^*rearr*^ ALL mostly leading to hematopoietic stem cell transplantation in first or second remission^30–32^. In line with this notion, Zaliova and colleagues have recently described a *DUX4* overexpressing subgroup that showed a poor molecular response to therapy^50^. Larger prospective studies are needed to determine the prognostic impact of DUX4^rearr^/ERG^alt^ in ALL.

From a technological perspective, gc-HTS might advance the clinical diagnostics of MRD by precise identification of genomic breakpoints and non-captured genomic partner genes such as RCSD1 or FOXP1, which is necessary for treatment stratification for instance of ABL-class (genomic) fusion-carrying ALL^51,52^. Using gc-HTS in the *BCR-ABL1*-positive ALL group we observed rare failures in the identification of genomic breakpoints that were potentially due to AT-rich- or repetitive sequences at probe hybridization sites or gaps in probe design targeting intron 1 of *ABL1*. Improved probe design with greater coverage at these sites may eliminate such drawbacks in the future.

In summary, the PCR-independent genomic approach presented here constitutes a very sensitive and robust method that enables laboratories to detect a broad spectrum of markers relevant for MRD diagnostics and also for targeted therapeutic intervention for instance in Ph-like ALL. A bespoke selection of genomic targets, a flexible workflow and the requirement for only very small amounts of diagnostic material should make this method widely applicable.

## Supplementary information

Supplemental material
