# Supplementary material for: Characterization of novel, recurrent genomic rearrangements as sensitive MRD targets in childhood B-cell precursor ALL
Source: Blood Cancer J. 2019 Nov 29;9(12):96. doi: 10.1038/s41408-019-0257-x (PMC6884523; doi:10.1038/s41408-019-0257-x)

Zur Stadt et al.

**Suppl. Table 1**

Summary of capture probes used for the current genomic capture assay (hg19)

| Target Region | Upstream Bases | Downstream Bases | Chromosome | Start | Stop | Selected Targets | Total Targets | Target Type | Total Probes |
| --- | --- | --- | --- | --- | --- | --- | --- | --- | --- |
| ABL1 | 1000 | 1000 | 9 | 133588268 | 133764062 | 1 | 1 | FullRegion | 1465 |
| ABL2 | 0 | 0 | 1 | 179068462 | 179198819 | 1 | 1 | FullRegion | 1087 |
| CRLF2 | 10000 | 10000 | Y | 1254887 | 1291530 | 1 | 1 | FullRegion | 306 |
| CSF1R | 0 | 0 | 5 | 149432854 | 149492935 | 1 | 1 | FullRegion | 501 |
| EPOR | 10000 | 10000 | 19 | 11477881 | 11505018 | 1 | 1 | FullRegion | 227 |
| IGH_JH | 0 | 0 | 14 | 106328000 | 106350000 | 1 | 1 | FullRegion | 184 |
| IKZF1 | 0 | 0 | 7 | 50344378 | 50472798 | 9 | 9 | Exon | 58 |
| IKZF2 | 0 | 0 | 2 | 213864411 | 214016333 | 11 | 11 | Exon | 88 |
| IKZF3 | 0 | 0 | 17 | 37913968 | 38020441 | 10 | 10 | Exon | 89 |
| IL7R | 0 | 0 | 5 | 35856977 | 35879705 | 8 | 8 | Exon | 29 |
| JAK1 | 0 | 0 | 1 | 65298906 | 65432187 | 25 | 25 | Exon | 55 |
| JAK2 | 0 | 0 | 9 | 4985245 | 5128183 | 1 | 1 | FullRegion | 1192 |
| JAK3 | 0 | 0 | 19 | 17935593 | 17958841 | 24 | 24 | Exon | 59 |
| JK | 0 | 0 | 2 | 89150000 | 89200000 | 1 | 1 | FullRegion | 218 |
| P2RY8 | 0 | 0 | Y | 1531466 | 1606037 | 1 | 1 | FullRegion | 622 |
| PDGFRB | 1000 | 1000 | 5 | 149492402 | 149536422 | 1 | 1 | FullRegion | 367 |
| SH2B3 | 0 | 0 | 12 | 111843752 | 111889427 | 1 | 1 | FullRegion | 254 |
| TRB | 0 | 0 | 7 | 142470000 | 142500000 | 1 | 1 | FullRegion | 131 |
| TRD | 0 | 0 | 14 | 22891000 | 22936000 | 1 | 1 | FullRegion | 196 |
|  |  |  |  |  |  |  |  | Total probes | 7128 |

**Suppl. Table 2: Sample UPN29: Comparisoin of MRD targets identified at Dx and at relapse and MRD values from from EOI.**

Targets were identified either by PCR and/or gc-HTS; the last 3 (stable) markers were only detected by gc-HTS

| **Method** | **target diagnosis** | **QR** | **MRD_EOI** | **Relapse (2½ y)** |
| --- | --- | --- | --- | --- |
| PCR | Vγ4-Jγ1.3 [-3/5/-5] | 1 x 10^-4^ | 5 x 10^-3^ | lost |
| PCR | Vγ4-Jγ1.3 [--8/9/-14] | 1 x 10^-4^ | 4 x 10^-3^ | lost |
| PCR/gc-HTS | Vβ20-Jβ2.3 [-4/7/-24] | 1 x 10^-4^ | 1 x 10^-2^ | 4 x 10^-1^ |
| gc-HTS | 7365 bp 5'-VH7*34 - JH6 | 1 x 10^-4^ | 2 x 10^-2^ | 7 x 10^-1^ |
| gc-HTS | ELK2AP-JH1 | 1 x 10^-3^  (b+) | 2 x 10^-2^ | 9 x 10^-1^ |
| gc-HTS | Dδ2-Jα09 [0/6/-5] | 1 x 10^-4^ | 2 x 10^-2^ | 9 x 10^-1^ |
|  | | |  |  |

**Suppl. Figure 1: Genomic organization of the capture TRδ region.**

IGV based screenshot shows examples from different type of rearrangements.

The –Jα61 positive sample consists of two clones (Vδ2-Jα61 and Dδ2-Jα61), with a common Dδ2-Jα61 stem.

RQ PCR assays were established for patient specific quantification of MRD follow up samples; number of positive Jα segments.


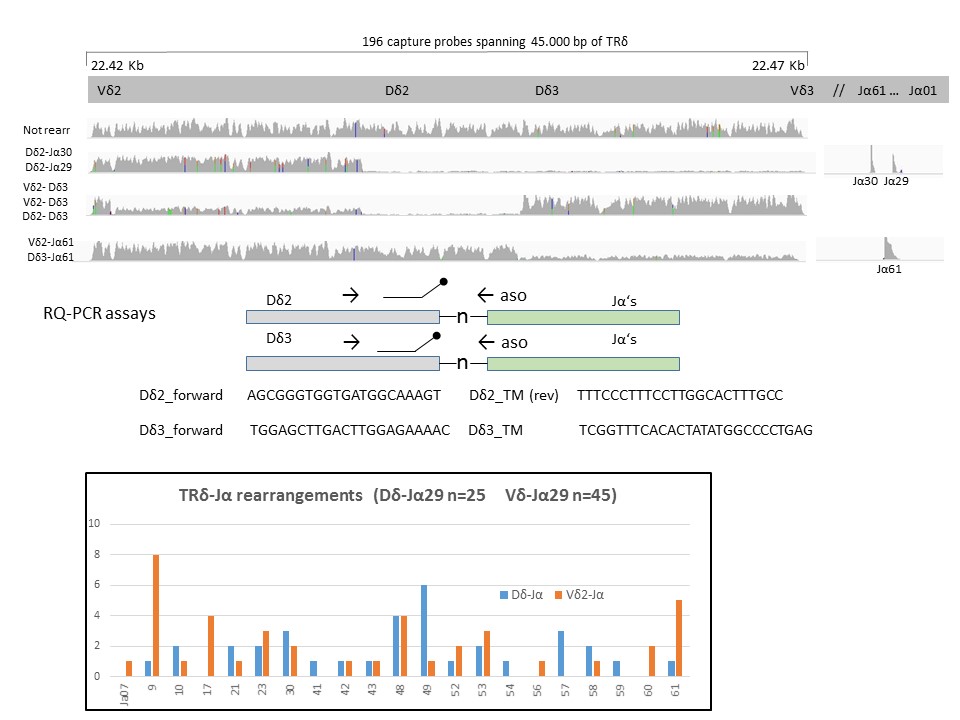


**Suppl. Figure 2:**

FOXI3 relative expression in B-cell precursor ALL as measured by TM assay with a LC480 machine.

Only weak to negative expression of FOXI3 was observed in FOXI3-IGH negative cases.

Case UPN22 served as a positive control.


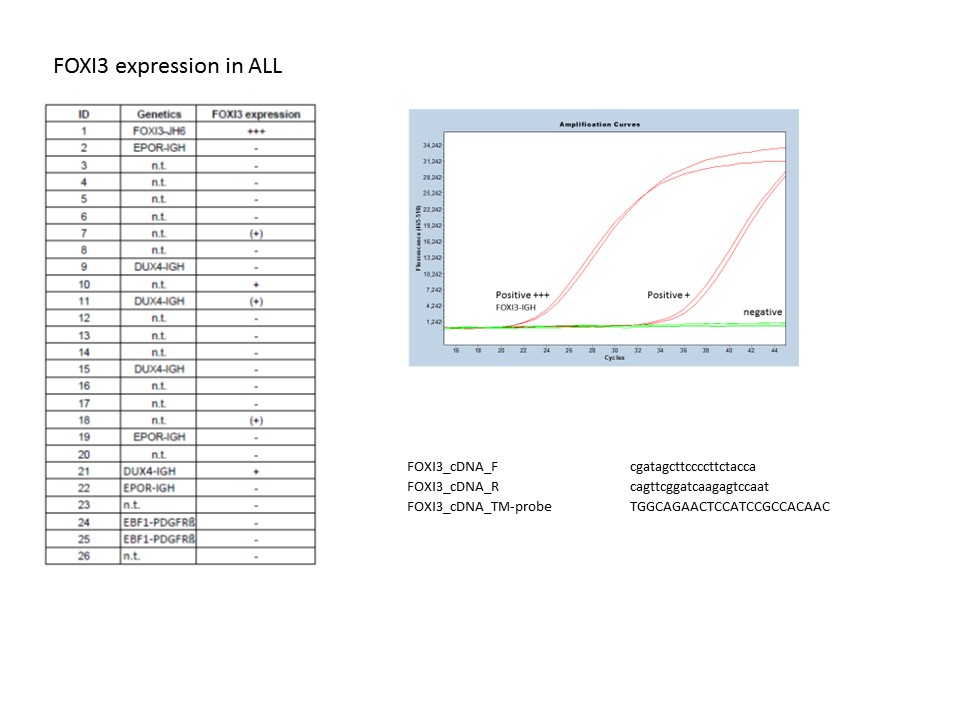


**Suppl. Figure 3: Genomic organization of the captured IGH-JH region.**

VDJ as well as DJ rearrangements were detected with the gc-HTS approach. The genomic structure of the IGH-JH-genomic fusions

are described in the lower part of the figure with the number of cases positive for each type rearrangement.


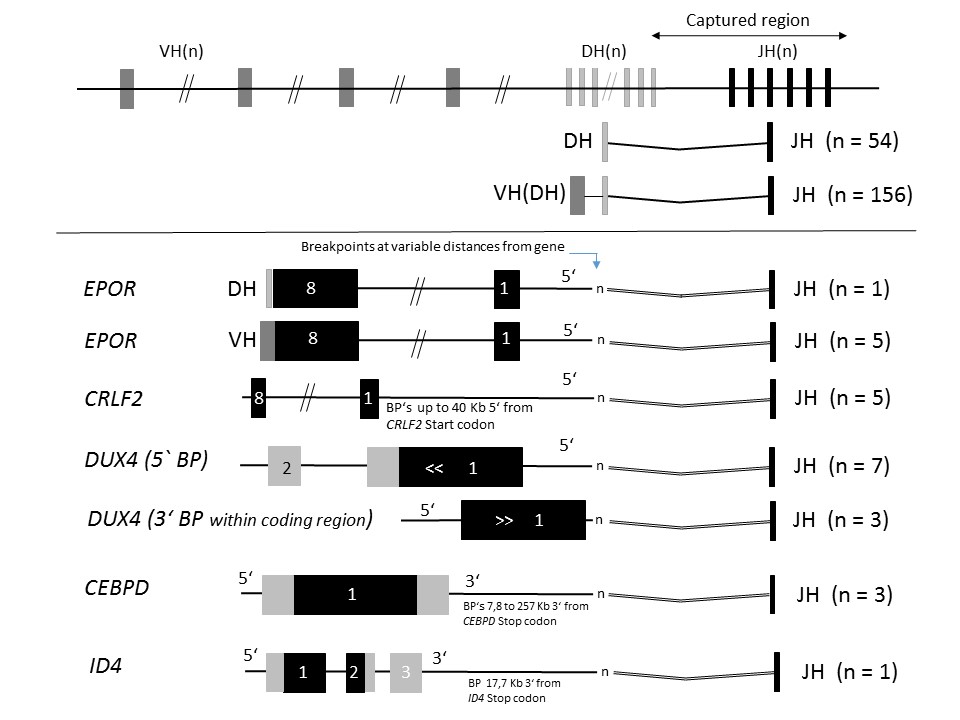


**Suppl. Figure 4:** Raw data from two different genomic fusion breakpoints.

IGV screenshots and the corresponding PCR based Sanger Sequencing results are shown.


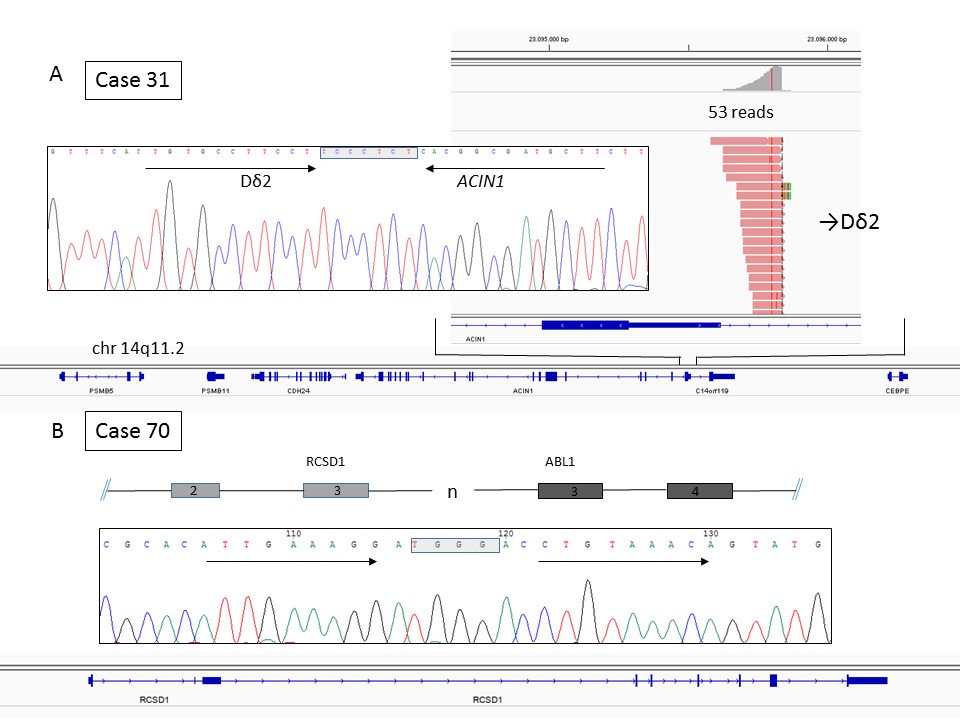


**Suppl. Fig 5:** UPN case26 with a genomic CTNNA3-IGH fusion. In addition to the genomic rearrangement a clonal V(D)J rearrangement

3 TRδ clones were sequenced.


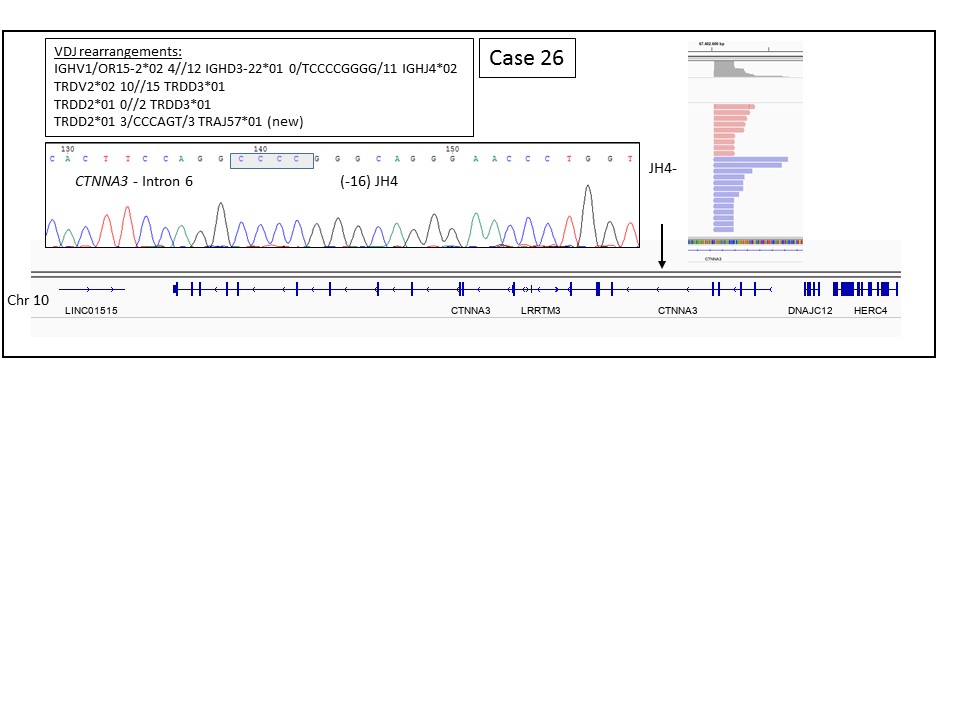

Supplement: Supplementary file 1 — Supplemental material [file 41408_2019_257_MOESM1_ESM.docx]
